# Former access to medicine higher education diploma students’ experiences of their diploma course and subsequent professional degree: a qualitative study

**DOI:** 10.1186/s12909-025-07399-x

**Published:** 2025-07-01

**Authors:** Samuel Moffatt, Ibrahim Inzarul Haq, Andrea Laczik

**Affiliations:** 1 Department of Surgery, University of KwaZulu-Natal, Durban, South Africa; 2 Department of Education, University of Oxford, Oxford, UK; 3 Department of Vascular Surgery, University Hospitals Leicester NHS Trust, Leicester, UK; 4 School of Psychology and Vision Sciences, University of Leicester, Leicester, UK; 5 Department of Orthopaedic Surgery, University of Cambridge, Cambridge, UK; 6 University Hospitals Coventry and Warwickshire NHS Trust, Coventry, UK; 7 Edge Foundation, London, UK

## Abstract

**Introduction:**

Access to Higher Education Diplomas (Medicine) are courses offered at UK Further Education colleges for mature students who lack the qualifications required to study medicine, dentistry or pharmacy). These courses are frequently referred to as Access to Medicine Courses (ATMCs) and provide students with the necessary entry level qualification to study these subjects at university. ATMCs play a role in efforts to widen participation in medicine for non-traditional students. However, relatively little is known about the experiences of former ATMC students during their course, professional degrees and early careers. This study investigates the experiences of former ATMC students who are practising doctors or pharmacists with a focus on decision-making surrounding the ATMC, positive and negative experiences resulting from being an ATMC student both before and after qualification, and their views and experiences of widening participation (WP).

**Method:**

Fifteen participants who had graduated from medicine or pharmacy after using an ATMC as their entry qualification were recruited using convenience and snowball sampling. Semi-structured interviews were conducted and the data analysed using thematic analysis.

**Results:**

Fourteen medical doctors and one pharmacist were interviewed. Ten participants were female and five male. Participants attended four ATMCs before studying at five universities. Nine participants could be classed as WP students. Interviews ranged from 30 to 75 min. Several themes and subthemes emerged from the analysis. The location of the course provider was a factor in ATMC choice. Reported disadvantages of ATMC were the limited number of medical schools accepting the qualification and deficiencies in chemistry. Advantages included supportive peers and tutors. Participants spoke about their experiences and views of WP and the role ATMC could play in it. Participants commented on the role that socioeconomic background and schools have in potential candidates aspiring to a career in medicine.

**Conclusion:**

ATMCs are crucial in efforts to widen participation in medicine, pharmacy and dentistry. There are issues with the courses that need to be addressed especially in how well they prepare students for medical school in chemistry. This research suggests that addressing shortcomings in potential candidates’ aspirations due to their socioeconomic backgrounds and schooling would increase the number of people applying to medical school from non-traditional backgrounds.

**Supplementary Information:**

The online version contains supplementary material available at 10.1186/s12909-025-07399-x.

## Introduction

### Background

It has been widely recognised that there is a requirement to widen participation in medicine. There are benefits to widening participation (WP) in medicine for reasons of social justice and because it may benefit patients to have a more varied group of doctors serving their needs [1–6]. Traditionally, doctors come from similar backgrounds. Of the foundation year one doctors surveyed in the 2014 General Medical Council (GMC) national training survey, 31% attended independent or fee-paying schools, despite only 7% of children in the UK being educated in these establishments [7]. Furthermore, work by Nottingham Medical School indicates that 80% of applicants to medical schools come from 20% of UK schools [7]. Approximately 75% of applicants to medical school have parents in higher managerial or professional occupations [8] and research suggests that students from lower socioeconomic groups were less likely to consider themselves able to study medicine [9]. In 2015 only 14% of new medical students were from lower socioeconomic groups despite these groups making up 56% of the general population [10]. The issue is best summarised by a quote from Milburn from their review on social mobility and child poverty which said: ‘it [medicine] has a long way to go when it comes to making access fairer, diversifying its workforce and raising social mobility.’ [11].

Access to Higher Education (HE) Diplomas are educational courses that act as an entry level (level three) qualification to a higher-level course at university [12] and have existed in some form in England and Wales since the 1970s [13]. They were initially established to allow non-traditional students to become teachers and were considered both a way of WP in general higher education and as a vehicle for social mobility [14]. The Access to HE Diploma (Medicine) is the specific course that acts as the entry qualification for mature applicants (without the required A levels or equivalent qualifications) to gain entry to university to study medicine (and either dentistry or pharmacy). These courses are often referred to as an Access to Medicine course (ATMC). ATMCs provide an atypical route into a career in medicine and are considered part of efforts to widen participation by their regulatory bodies the Medical Schools Council (MSC) and the Quality Assurance Agency for Higher Education (QAA) [15, 16]. 

The MSC (2020) describe ATMCs as:

*‘a route into medicine for mature learners who do not possess the usual formal qualifications*,* such as A Level Biology and Chemistry*,* or equivalent. They are delivered in further education colleges and are targeted at those who plan to attend college after an extended period out of full-time education.’* [15].

ATMCs are generally one year in length and differ from foundation courses which are also integral to WP [17]. There has been very little published literature about ATMCs with a comprehensive search resulting in only two results specifically mentioning them.

The first result was a 2002 report on an ATMC being run by the College of West Anglia in King’s Lynn, which appears to be one of the longest running ATMCs in the country [18]. This report primarily describes the first three year groups that completed the course and their progress through medical school. The report also describes the establishment of the course and its structure. This report is a small-scale quantitative study and reports on student outcomes, but it is limited in what it contributes to the understanding of students’ experiences because this is not what it was designed to investigate.

The second result was a thematic analysis of data collected during in-depth unstructured interviews with 12 mature medical students from working-class backgrounds [19]. This research was not specific to ATMCs, but five of the 12 participants had an ATMC as their entry qualification to study medicine. This research explores the students’ experiences (albeit not specifically because of their being ATMC students, but due to their socioeconomic backgrounds). Participants in this research described key areas of their decision making around entering into medicine as mature students and the role that their schools, educational environment and social background played in this. Limited exposure to medicine, low educational expectations and lack of support whilst at school were key factors in participants not deciding to pursue medicine at the conventional age (around the age of 18 years old).

## Method

Recruitment of participants was initially via a convenience sampling method, followed by an exponential non-discriminative snowballing sampling method. The inclusion criteria were post-qualification doctors, dentists or pharmacists who had used an ATMC qualification to gain admission to university for their professional degree. These inclusion criteria were chosen to investigate participants’ experiences; before, during and after the ATMC, through their professional degree program and into their early careers.

Semi-structured interviews using pre-prepared questions were conducted with participants, in person or via Microsoft Teams (according to participant preference, so as not to hinder recruitment) by SM. Interviews were recorded before being transcribed using Microsoft Word’s automated transcription function. Post-transcription the texts were assessed against the recordings to ensure accuracy and then anonymised (with the recording being deleted afterwards). Notes and memos were taken at the time of the interview and immediately afterwards. The pre-prepared interview questions are shown in Supplement 1. No software was used in coding, with manual coding of the data being conducted by SM, with AL acting as supervisor.

### Analysis

Analysis of interview transcripts, memos and field notes was conducted using thematic analysis [20] to address the following research questions.

A. What factors influence ATMC students’ decisions to pursue medicine, dentistry or pharmacy and their choice of ATMC?
B. What are ATMC students’ experiences of their access course, professional degree and early careers?
C. What are ATMC students’ views on and experiences of WP?

### Ethics

#### Ethical approval

for this research was granted by the University of Oxford CUREC – reference R84473:RE001. This research adhered to the British Educational Research Association’s ethical guidelines for educational research [21]. Informed consent to participate was obtained from all participants included in this research.

### Positionality statement

The lead author (SM) attended an ATMC at Sussex Downs College from 2011 to 2012 and used this qualification to gain entry to medical school. IH and AL do not have any personal investment in ATMCs.

## Results and discussion

### Interview sample

Fifteen participants were interviewed between February and April 2023. Two interviews were conducted in person and 13 via Microsoft Teams (due to participant preference). Participants had attended ATMCs at four colleges and went on to attend five universities. Five of the participants identified as male and ten identified as female. Fourteen were practising doctors and one was a practising pharmacist. One participant had completed their training as a GP, and the pharmacist had completed their training. The remaining doctors were still in postgraduate training or trust grade roles (a trust grade doctor is employed locally by an NHS trust and is not in a formal training role).

The age of the participants on starting the access course ranged from 22 to 43 years old, with a median age of 31 years old. Nine participants fulfilled criteria to be classed as WP students due to being the first generation in their family to attend university (*n* = 8) and based on their race (*n* = 1). Highest-level qualifications before starting the ATMC varied considerably between participants. The highest-level qualifications held at the time of applying for the AMTC were; A-levels (*n* = 2), a higher educational diploma (*n* = 1), a BTEC national certificate (*n* = 1), professional insurance underwriting exams (*n* = 1), bachelor’s degrees (*n* = 7), master’s degrees (*n* = 2) and one participant was in the process of submitting their PhD thesis.

Previous occupations (prior to ATMC application) included: a radiographer, four nurses, a midwife, a student, a teacher, an insurance underwriter, a restaurant manager, a clerical worker, a builder/antiques dealer, a banking consultant, an information technology operative and an NHS direct call centre operative. Twelve participants had paid healthcare work experience prior to starting the ATMC. Of the remaining three participants, one had done voluntary work at a homeless shelter, and one had some unpaid work experience in a healthcare setting. Only one participant had no prior healthcare work experience at the start of their ATMC.

A total of 12 h, 53 min and 54 s of interviews were recorded. Interview times ranged from 30 min and 50 s to 1 h, 15 min and 56 s. The mean interview time was 51 min and 36 s. Participants have been assigned pseudonyms in this paper.

### Analysis

Four main themes emerged from the analysis, each with a number of subthemes, which are displayed in Table 1.

**Table 1:** Themes and subthemes

Theme	Sub theme
**Decision-making about the ATMC**	• Reasons for choosing to study medicine or pharmacy as a mature student • Reasons for choosing to do the ATMC versus A-levels • Reasons for choosing a specific ATMC • The role of location in choosing an ATMC
**Disadvantages of the ATMC**	• Academic position compared to traditional route students • Inadequacy of ATMC chemistry • Restricted number of medical schools accepting the ATMC
**Advantages of the ATMC**	• ATMC as an entry-level qualification to a professional degree • Peer support • Tutors • Applying for medicine – personal statement • Post-qualification - impact of gaining entry to professional degree via ATMC • Academic benefits of the ATMC • Benefits of being a mature student studying for a medical/pharmacy degree and post-qualification
**WP and ATMCs**	• The influence of school and socioeconomic background on people applying to study medicine • Flaws in the current UK medical school selection process • Advantages of WP • Difficulties in WP • Stigma of being a non-traditional route student during professional degree

### Decision-making about the ATMC

#### Reasons for choosing to study medicine or pharmacy as a mature student

All participants commented on their reasons for deciding that they wanted to pursue medicine at a later stage in life (the participant who was a pharmacist initially enrolled on the ATMC to pursue medicine). Many of the participants had not known what they wanted to do career-wise in their late teens and early twenties and described entering into professions because opportunities arose, rather than making a conscious decision to pursue a particular career path. Other participants had not chosen to pursue the sciences at A-level because they had not known that they wanted to study medicine. Several participants eventually found themselves working in healthcare settings, and this exposure was significant in their decision-making around wanting to study medicine:

There were questions that I had about the patient encounters on the telephone, at NHS Direct and this resulted in me spending a fair bit of time just looking up certain conditions, ailments that people mentioned that had….

…I don’t know, I wanted to know a little bit more. More than anything else. (Joe)

For another participant, significant life events prompted them to act upon thoughts that they had been having for several years about the possibility of studying medicine:

I’d lost my dad who died in 2009, and I’d taken six months off to look after him as his, as his primary carer; that’s quite a profound experience and the chance to step back, look at things. Look at the way going forward, and that’s really when I decided, I’m going to go for this, it is what I wanted to do. (James)

The motivations of the participants were varied but are in keeping with other literature investigating mature students’ decisions to study medicine. Mathers and Parry found that some mature medical students had a longstanding interest in medicine whilst others developed this interest later through exposure to medicine in a number of settings, including the workplace [22]. Interestingly, the majority of the participants in that study had some aspect of ‘self-fulfilment’ driving their decision to study medicine, and this was also demonstrated by most of the participants in our research.

### Reasons for choosing to do the ATMC versus a levels

Participants decided to apply to the ATMC rather than A-levels for four main reasons:

I. They thought that the ATMC would offer better personalised support, increasing their chance of academic success.
II. Participants’ concerns about the much younger peer group associated with studying A-levels.
III. A-levels would be more expensive (with less opportunity to do additional paid work).
IV. The ATMC would offer the quickest route into medical school (being a one-year course rather than two years with A levels).

### Reasons for choosing a specific ATMC

At the time that participants applied to their respective ATMCs the number of colleges running them nationally was low.

There didn’t seem to be a whole… huge number of Access to Medicine Courses out there really, but certainly they were the only two [Sussex Downs College and College of West Anglia] that really stood out to me. (Lucy)

Several of the participants had applied to more than one ATMC, with the courses at Sussex Downs College and the College of West Anglia being the most prominent courses running at that time. Two major factors in participants’ choosing their access course were how well linked the course was with a medical school/s and their evaluation of the likelihood that the access course would help them gain admission to medical school. At the time when participants applied to the Sussex Downs College ATMC, it had strong links with the local medical school (Brighton and Sussex Medical School). Candidates who were accepted onto this access course were given an interview at the medical school in addition to the normal Universities and Colleges Admissions Service (UCAS) application process, which is known as a linked interview [15]. UCAS is the centralised body in the UK which applicants apply to universities through. Just under half of the participants interviewed described close links with medical schools as a factor influencing their decision to apply to a particular ATMC. Participants also had to consider that ATMCs were only accepted as an entry-level qualification by a limited number of universities (this is described further in the negatives of the ATMC section).

### The role of location in choosing an ATMC

The locations of the colleges that provided the ATMC were extremely important to participants. Thirteen participants reported this as a factor in their decision-making when considering which course to apply for. There were several reasons that location had an impact on the choice of ATMC. These included; life and family commitments preventing the participant from moving to attend the ATMC, the financial implications of moving away from an area and leaving paid work behind, loss of their usual social support network and concerns about relocating if progression beyond the ATMC was uncertain. One participant said:

So, when I was initially applying for the access course, I don’t know that I would have done that if it hadn’t have been that there was something local to me. (Emma)

Participants who were located within a commutable distance of the ATMC described this as being a major factor in deciding to attend the course, and some may not have decided to attend the course if it were located further away. The financial benefits of being able to remain in a location that was a commutable distance from an ATMC provider and retain paid work (versus having to relocate and the potential loss of earnings) were a significant factor in some participants’ decision to apply for a specific course:

I needed a location that would allow me to work and earn money and also travel easily to. A location where I could do the… attend the course and then have enough time to do the study and the course work that was prescribed for us. (Lucy)

Other significant reasons that participants gave for not being in a position to relocate to study an ATMC were; that they owned a house in the area they were living and would not have been in a financial position to move and that they were involved in long term relationships which would prevent them moving away from where they were based.

Our study demonstrates that location is a significant consideration in ATMC choice. This presents challenges for potential candidates attending an ATMC, which could negatively impact WP efforts if such courses are not easily accessible in their area. The number of ATMCs has increased substantially since our participants attended them (from a handful to 26) which should open this route to more WP candidates [23]. 

### Disadvantages of the ATMC

#### Academic position compared to traditional route students

There were two main views expressed about how well prepared ATMC students felt when starting their professional degrees (medicine or pharmacy), if they compared themselves to their traditional route student peers. These views were split equally between feeling as well prepared as A-level students and feeling poorly prepared. Some participants felt very underprepared for their professional degree:

I think I did not get a good educational experience on the access course and I think obviously I did enough to like get into medical school. But I felt so woefully unprepared when I got to med school….

…I remember we had in the first week there was a physiology lecture and they were talking about something, some like feedback thing [a physiological regulatory mechanism] and I remember thinking what the [profanity] is going on? And then the lecturer said OK, turn to the person next to you and fill out like these gaps and so I turned the person next to me who I didn’t know, and I expected them to go what is this about? And they were just like oh so I guess it would be blah, blah, blah, blah, blah, and I remember having a moment of just like oh my God, other people actually understand what’s happening and that moment of realisation. (Ruth)

Another of the participants also felt that they were not as well prepared for their professional degree (in this case medicine) compared to their A-level colleagues. They thought that this was the result of having to cover a lot of material in a shorter time and not due to any issues with the quality of teaching:

I don’t think I felt as academically well prepared as I suspect I would have been if I’d gone and done the A-levels as an eighteen-year-old and come straight through. I think that wasn’t specifically the access course’s fault, but you’re doing it in one year on the access course. We covered five or six subjects inevitably, in less depth, I think. (James)

### Inadequacy of ATMC chemistry

There was a feeling amongst some participants that they were not well prepared for their professional degrees by the ATMC. One particular area that came under criticism was ATMC chemistry. Six participants made specific reference to the ATMC chemistry being at an inadequate level for starting their professional degrees. ATMC students described having to try and catch up with chemistry for their professional degrees.

Well, like I mentioned, the chemistry, not having formal [A-level] chemistry was difficult and I had a lot of catching up to do the first year at med school. (Rob)

Another example of a participant feeling underprepared at medical school because of inadequacies in ATMC chemistry is shown below. There is some similarity to the example given by Ruth in the previous section (although this experience is more specific to chemistry). Emma and Ruth attended different ATMCs and different medical schools.

When I got to medical school and we sat down to do like the biochemistry, like particularly one thing, the molecules, genes and diseases module. That was like the biochemistry, like the hardest thing of the whole course for me, because it was like A-level chemistry stuff and we hadn’t covered that to that degree, which I don’t think you could expect it to, to be honest. But like, you know, the people I was with in my little group, I remember being at the table and they’ve got all these DNA models out and it was like easy-peasy to them and I just felt like, oh, my gosh, you know. It made me feel at that time on that day, is this the right place for me? Am I in over my head, and for that one particular module, I used to just have to say to myself, don’t worry about it. It’s just one aspect, and just don’t let it get you down, and that was the only time I’ve ever felt like that. (Emma)

Whereas in the subtheme – academic position compared to traditional route students, there was a mix of experiences, when reflecting specifically on ATMC chemistry, no participant described feeling well prepared for their professional degree.

### Restricted number of medical schools accepting the ATMC

At the time that the participants undertook their ATMCs the qualification was only recognised at a limited number of universities. Eight of the participants described this as being a disadvantage of the ATMC. One participant was particularly disparaging of university admission policies surrounding the ATMC at that time:

So, one obvious negative for me was that the access course isn’t accepted by every single university, so obviously that’s stupid, but you know, if you do A-levels, you’ve got a lot more universities to apply for. (Becky)

The disadvantage of the ATMC being accepted by a small proportion of universities was compounded for the one participant included in this research who did graduate entry medicine (in the UK the majority of medical degrees are five year long undergraduate courses, but graduates can apply for a shortened four-year medical degree course at some universities). They described having only three or four graduate medical courses that recognised the ATMC nationally. At roughly the time that the participants included in this research were applying to medical school 22 universities accepted the ATMC (mostly for entry to undergraduate medical degrees but this figure also includes the graduate entry courses just mentioned) [24]. As of 2023 the MSC website and individual medical schools’ websites show that this figure remains at 22.

### Advantages of the ATMC

#### ATMC as an entry-level qualification to a professional degree

The main advantage of the ATMC described by the participants, was that it had been the entry-level qualification for their professional degree. This was the case for all participants except for one, who was questioning their decision to become a doctor and felt undervalued and unhappy with their current work conditions. The role of the ATMC in securing participants a place on a medical or pharmacy degree was summarised by Lucy, who said, ’you know it did what it said on the tin.’

### Peer support

The most frequently described benefit of the ATMC was the peer support. Fourteen of the fifteen participants interviewed spoke about the peer support on the access course as being a major advantage. This peer support came in several forms ranging from the general environment on the ATMC through to peers supporting each other academically if someone was struggling with a particular subject or concept:

I think because everyone was in the same boat, like I mean there, well, there were people on the course obviously from huge, you know, variety of backgrounds and previous experiences, and I think we all just helped each other along. And if in those particular assignments one of us, you know, were struggling or vice versa, we could help each other along. (Amanda)

Having a common goal was described as a key component of the camaraderie felt between ATMC students. Several participants commented that they did not feel as if they were in competition with their peers on the ATMC. One participant described this as being in contrast with the atmosphere at their medical school, where people would be reluctant to disclose educational resources to avoid giving their perceived competitors any advantage. An interviewee suggested that the positive culture amongst peers on their ATMCs was to some extent due to the attitude and leadership of the staff member leading the course.

### Tutors

Eight participants spoke highly of their ATMC tutors. They described that the tutors worked extremely hard to support them and they went above and beyond what might be expected for their given role. Participants described how the access course tutors were happy to provide extra support and to be available to meet or help students:

I did have very good support from the course tutors, often times one-on-one support, a lot of times out-of-hours one-on-one support, so I think the lectures and the course leader worked very hard indeed to do everything they could for us to ensure that we got the most out of it. (David)

Interviewees felt that the ATMC tutors were heavily invested in seeing students gain a place at university and really wanted their students to succeed: ‘They were willing us to do well, they were quite exceptional in that regard’ (Sarah). One participant offered the opinion that the ATMC format had a role to play in the tutors working so hard to ensure that students got the required distinction level grade to progress onto their chosen professional degree. They thought that this was due to the small size of the course and the very specific aim, which meant that tutors would get to know their students and know exactly what they were working towards. They speculated that this would not be the case on a normal college A-level course.

### Applying for medicine – personal statement

Ten participants spoke about the advantage that they gained from the ATMC with the UCAS personal statement for their medical school application. The course tutors gave guidance on writing these statements and reviewed them prior to submission. The ATMC tutors had expertise from assisting in writing personal statements for numerous access course students per year - with (in the author SM’s experience) the majority gaining places. Reflecting on the help they received with their personal statement, one participant said:

I remember the personal statements being reviewed as part of the course, and I can remember getting feedback, and I think that was quite rigorous. You know, several times it was too long and I remember finding that that useful and feeling that yes, actually this has improved it. I think I thought, oh, I’m a writer and I’ve got a background in PhDs and literature and that kind of thing. I thought that’s something I would be good at, maybe I was fairly good at it, but I definitely got value added from the course, and the main person running the course was the one who helped with that, and that was good. (James)

### Post-qualification - impact of gaining entry to a professional degree via the ATMC route

Participants generally felt that post qualification, the route that they took into medicine or pharmacy has had no negative impact whatsoever. Eight participants specifically said that being an ATMC student has had no negative impact on their professional career, and none of the remaining participants described any negative impact on their career or career progression.

### Academic benefits of the ATMC

The main academic benefit of the ATMC was that it gave interviewees the opportunity to become familiar with or refamiliarise themselves with the skills associated with studying. Although all participants had some experience of post-GCSE level education, most had not undertaken any formal learning for several years or had no experience of studying the sciences post-GCSE. Interviewees described feeling that the ATMC was a good reintroduction to education and that it helped them refine their time management skills and study techniques. One participant who had not previously studied science past GCSE level described the access course sciences as starting at an appropriate level and progressing at a pace that was manageable. Other participants said that the ATMC improved their academic confidence.

Mathematics and statistics were frequently mentioned by participants as subjects taught to a particularly high standard. One participant noted that the strong foundation they gained from the access course enabled them to help their traditional route peers at medical school who faced difficulties in these areas:

We did a year of higher maths there as far as I’m concerned. You know, I can still, I can still look at statistics and figure out what the [profanity] going on, and it’s entirely based on that [Access to Medicine Course statistics] because what they taught me in medical school was a little bit rushed and clunky and yeah, like I said, I ended up kind of coaching other very bright people through that part of medical school, so I had a good handle on it. (Rob)

### Benefits of being a mature student studying for a medical/pharmacy degree and post-qualification

The criteria for admission to ATMCs mean that all students are mature students. The participants generally felt that being a mature student and having life experience offered them some advantages, especially in the clinical setting (both as students and post-qualification). Participants who had found the non-clinical phase of their medical school education challenging found that they were much stronger in Objective Structured Clinical Examinations (OSCEs) and the clinical phase which they attributed to improved communication skills that they had developed in previous job roles or through prior life experiences:

When we got further through medical school and we were into the clinical side of things, the maturity factor really kicked in and we were flying through our OSCEs and flying through the, you know, the real clinical stuff. Whilst I know a lot of the younger guys were struggling a bit. (Jenny)

Some of the participants were also able to draw on previous experiences of their working lives before their post-qualification job role to give them resilience when work was difficult. There were also several references to being more appreciative of the job role they were currently in, due to the path taken to get there.

### WP and ATMCs

#### The influence of school and socioeconomic background on people applying to study medicine

Several participants spoke about the impact that schools and socioeconomic background had on their decision to study medicine. The majority of these participants spoke about not having had medicine or the sciences suggested to them at school or about attending a school where university attendance was not seen as a realistic option.

The school I went to, I don’t think they were looking at churning out doctors. (Rob)

One participant did attend schools that made them feel like medicine was open to them as an option. These schools were fee-paying schools. They said:

Yeah, so to speak, in person I feel fairly like in a privileged position, in that the kind of schools that I went to, maybe that made me more confident to do this sort of thing. To think that I could do medicine fullstop, maybe it gave me that. (Amanda)

Another participant questioned if the current state of the educational environment and funding favoured those from a wealthy background.

I think a lot of doctors do seem to come from particular types of backgrounds, to be very privileged, very wealthy, perhaps you need to be these days to get through. (James)

Participants also spoke about there not being an aspirational environment in their school or socioeconomic background. One participant describes not belonging to the ‘tribe’ of people at their school who went on to study medicine at university and felt written off by one of their teachers. The role of socioeconomic background in determining aspirations towards higher education is summarised by the following statement by a participant talking about the area that they grew up in and the views held about higher education:

Yeah, it’s well, you know, Merseyside, very rough, very impoverished area. My parents, my Dad was a docker and my Mum was a nurse, and they, you know, learned on the job, and higher education was for privileged people, not for us. So I was never, it was never even entertained that sort of level of education. (Sarah)

One participant spoke about how the quality of schools varies by location and the importance of recognising that schools have a vital role to play in making sure studying medicine is accessible to anyone who has the academic potential to succeed.

You might end up going to a lovely country school that has one teacher for 12 children, and you might end up in a city centre school that is one teacher for 30 children. So, it really is important, I think that it is recognised there is a huge disparity. (Lucy)

This comment by Lucy offers a compelling argument supporting the role of contextualised admissions in professional degrees such as medicine, dentistry and pharmacy.

### Flaws in the current UK medical school selection process

Participants expressed a view that the way medical schools select students is flawed. There was a suggestion that picking medical students based on their academic attainment in A-levels did not produce the best quality doctors (although it must be noted that other factors such a personal statements, interviews and additional tests such as the University Clinical Aptitude Test or BioMedical Admissions Test are used to assess applicants for entry into medical school) [25, 26].

You know, when you are in it, your credentials aren’t particularly scrutinised by your patient. Your bedside manner, how you communicate, your decision making, how you articulate and communicate and translate medical knowledge to a layperson. That’s not an A star in a GCSE or A-level that’s just being a person and just being good and wanting to be good at your role. (Hannah)

The role that WP might play in selecting students who want to work with patients rather than making selection choices based on A-level results was also highlighted:

So, I think actually one thing about widening access should be the driver… should be to get the right people to be doing medicine. The right people are the ones that want to work with patients not yeah, not necessarily the ones who can get 100% in a chemistry A-level. (Kate)

### Advantages of WP

All of the interviewees expressed their support for efforts to widen participation in medicine or pharmacy. The reasons for this support varied, but one area that was seen as an advantage of WP was to provide clinicians who were more representative of the populations that they served. Participants expressed views that this could improve the quality of the care that patients received, especially through having clinicians with a greater understanding of their patients and improved communication skills:

So, our country is diverse in certain senses and it would be good to have people from different communities as doctors in order to support people from different communities in society. (Joe)

There was also some degree of concern about social justice in that people wanting to pursue a career in medicine should not be disadvantaged by their background if they had the potential to pass the course. One participant said, ‘I think if someone’s got the capability, they shouldn’t be held back by their background’ (Elizabeth).

Several participants spoke about the role that the ATMC could play in WP. One participant made a comparison between medical school foundation courses and access courses in terms of WP, saying:

They’re probably equally positive things to have in the sense that they provide routes into medical training for people who otherwise would not have been able to have those opportunities. (David)

### Difficulties in WP

Participants did identify several problems with or obstacles to WP. These included: the requirement for more schemes to widen participation, the possibility of a higher dropout rate in WP students, the view that WP schemes are only worthwhile if the clinicians produced by those schemes stay in their professional role and that without adequate financial support, WP students might not be able to attend university. One participant pointed out the need to be realistic about how challenging medical school can be to give WP students a chance to be adequately prepared:

I think it’s just important to flag up how challenging that it is [medical school]….

…so that people from backgrounds which are different are able to realistically go into it and get through, because I think you know, there was quite a high dropout rate. (James)

The role of funding was also highlighted as an area that could potentially hinder efforts to widen participation:

You know, for the sort of people that you’d be looking forward to wide participation, particularly from, like lower socioeconomic groups. I think there would definitely be a [financial] struggle. And I think very few people would be. You wouldn’t be able to… that sort of amount, it’d be hard to self-fund if you didn’t have support. (Harry)

One participant stated that they did not like the idea of having quotas of people with certain characteristics, but that measures should be put in place to level the playing field:

I think forcing wider participation and having that focus is just all like it’s the wrong way around in my head. Like, I just never really understand it. To me, you just have to give everyone the same platform. (Becky)

Participants felt that efforts to widen participation should not be allowed to result in a reduction in the standard of clinicians being produced. One participant felt that their ATMC was not particularly diverse and several others expressed concerns that the ATMC route was not a particularly well-known way of gaining admission to study medicine or pharmacy and this could limit the usefulness of this route in WP.

### Stigma of being a non-traditional route student during professional degree

The former ATMC students interviewed had a mixed view of there being some stigma attached (whilst at university) to them having gained entry to their professional degree via a non-traditional route. Six participants felt that there was some stigma attached to being a non-traditional route student, whilst nine felt that there was not.

## Discussion

This study appears to be the only research that provides an insight into the experiences of ATMC students throughout their access course, their professional degree and some of their early careers. Participants had often ended up falling into other job roles after leaving education (both secondary and higher education), and then made the decision to pursue medicine after re-evaluating what they wanted from their careers. For many participants this came after a period working in healthcare or through some other healthcare exposure. This finding is consistent with research by Holmes that described 22 out of 26 ATMC students as having previously been employed in another healthcare role, such as nursing or the ambulance service [18]. Significant life events were also a reason that interviewees gave for their change of career direction.

The main factor why participants chose to do an ATMC rather than to do/redo A-levels was that they felt they would receive more personalised support on an ATMC and have a greater chance of success. The remaining factors were that they were concerned about having a much younger peer group if they did A-levels, that A-levels would take longer (two years in comparison to the one-year ATMC) and that doing A-levels would be more expensive with less opportunity to do some concurrent paid work. Participants chose their specific ATMC (from the handful running at the time) based on which universities would accept the course and the links that the colleges running the courses had with local medical schools. Location of the colleges running the ATMC was a major factor in the decision-making process for most of the participants. This was due to life and family commitments, financial reasons and wanting to remain close to social support networks. Previous research has demonstrated that location is a crucial factor in course or university choice for general Access to HE Diploma students [27–30]. 

ATMC students described a range of positive and negative experiences of the ATMC, their experiences at university studying medicine or pharmacy and their early careers. Many of the participants did not feel as well prepared for their degree program as the traditional route students and this was especially true for ATMC chemistry which was described as being insufficient by the majority of the participants, which is problematic because the Access to HE Diploma (Medicine) subject descriptors for medicine state that the course should be at A-level standard for chemistry [16, 31]. Further negatives of the ATMC included that it was not accepted as an entry qualification for all medical schools.

Positives of the ATMC were that it did provide a route for these participants to gain entry into their professional degree program and that the tutor and peer support were excellent. Many participants felt they benefited from help with their UCAS personal statements. The ATMC provided students with an opportunity to refamiliarise themselves with an academic environment and improve time management and study skills. Maths/statistics was one area participants felt was taught to a high standard. Many of the ATMC students felt that although they might have been at a disadvantage during the non-clinical part of their professional degrees, their life experience gave them an advantage in OSCEs and the clinical side of their undergraduate training. Post-qualification, none of the participants felt that the route they had taken into their job had an impact on their career progression.

All participants expressed support for efforts to widen participation in medicine or pharmacy. WP was seen as advantageous because having doctors who are representative of the population would improve the quality of care delivered to patients. Many of the participants also expressed support for WP due to social justice reasons. Overall, the participant’s views on WP echo the views presented in other research [32–34]. Dowell et al. conducted a survey of Scottish GPs and found that they were more likely to work in deprived areas if their parents had been employed in semi-routine or routine occupations versus managerial or professional occupations. They concluded that widening access could improve service delivery in deprived areas [32]. Girotti, Park and Tekian argue that WP schemes can diversify the medical workforce [33] and this view was also expressed by a participant in research by Cleland et al. who said ’if we can recruit students from the local area, they’re more likely to want to work here in future’ [34]. Our research adds further evidence supporting these findings. Several participants highlighted the role that ATMCs could play in WP. However, participants identified some issues with WP, including the need for adequate funding to support WP students and that the standard of clinicians at qualification should not be reduced. There is good evidence to suggest that widening participation does not reduce standards of clinicians in actuality with a 2021 meta-analysis of 41 medical schools’ assessments (both written and OSCEs) over a five-year period finding a small but statistically significant differences between WP and traditional students' results which the authors concluded was inconsequential in the real-world [35]. Participants also suggested that there were an inadequate number of WP schemes and that there may be a higher dropout rate amongst these students.

The role that schools and socioeconomic environment have in WP in medicine also featured prominently in the interviews. Several participants spoke about attending schools where participation in the sciences was not particularly encouraged, and these were not schools that encouraged students to think about careers in medicine. Existing literature demonstrates the role that schools play in students deciding to attend university, with a study of 277 schools in Wales finding that attending certain schools reduced the likelihood of university attendance by 80% [36]. Furthermore, it has been reported that sociocultural beliefs associated with socioeconomic status can influence habitus development so that people feel that higher education is not for them [19, 29]. The results of our study are consistent with this, and some participants spoke about coming from backgrounds where a university education was not thought of as being attainable. These results are also consistent with Bourdieu’s General Theoretical Framework [37–40] from the perspective of children from WP groups developing a habitus whereby they do not believe they are able to study medicine or pharmacy. Some participants felt that being a WP student could carry some degree of stigma but the majority felt that it did not. Some of the interviewees offered the opinion that the medical school selection system was flawed. They criticised the emphasis placed on academic achievement over other relevant characteristics and suggested that this does not help diversify doctors but contributes to the lack of representation of certain groups within the profession. Existing literature demonstrates that lower socioeconomic groups are poorly represented in medicine [7–11], and the views expressed by participants in our study are consistent with this.

There are a variety of areas that this research has served to generate further questions in. All participants interviewed for this dissertation were successful in passing the ATMC and their professional degrees. Research into why people accepted into an ATMC fail to complete it, or research on those who do pass it but fail to complete a professional degree, could be valuable in understanding how the factors causing this failure could be addressed. One area that this research has identified where improvements could be made is ATMC chemistry, which participants considered inadequate for starting a medical or pharmacy degree.

The participants spoke highly of the tutors who ran the courses, and efforts should be made to retain staff who are so committed to their roles. There is scant research into ATMCs, however, research into Access to HE Diplomas for other subjects has found that the tutors are very committed to helping students succeed. Busher, James and Piela interviewed Access to HE Diploma tutors (for subjects other than medicine) and reported that the tutors put a high level of effort into teaching on these courses compared to other teaching duties [41]. Additionally, our research highlights the benefit of having access to people who understand how to succeed in applying to very competitive university courses. This is demonstrated by the advantage ATMCs can provide in tutors having experience of helping with UCAS personal statements specific to medicine or pharmacy.

The supportive peer group on ATMCs was an area highlighted by this research as being incredibly important to interviewees, which was, in some cases in contrast to the peer group support at medical school or in the workplace post-qualification. Previous research into general Access to HE Diploma courses has also described the importance of the peer support they offer, describing it as a ‘sense of community’ or ‘community of practice’ [42]. The access course was a way for students to develop study skills and become familiar or refamiliar with the academic skill set required for their professional degree courses. Previous qualitative research by Hinsliff-Smith, Gates and Leducq using focus groups of Access to HE Diploma in Nursing students has shown that students develop skills throughout their course that enable them to meet the demands of the course itself and that they carry forward into Higher Education [30]. As mature students, ATMC students felt that they had a range of additional skills and advantages over their colleagues at university, and there were numerous examples given of these benefits continuing post-qualification.

### Limitations

This study has a small sample size and has some limitations. It was only able to recruit participants from four ATMCs. This was in part due to the study design recruiting participants who were post professional qualification (to explore some of the impact of the ATMC and background on their professional career). Consequently, at the time that these participants were applying to their ATMCs, there was a limited number of them running nationally. There are now many more in operation, with 26 specific ATMCs listed on the QAA website [23]. The decision to use convenience and snowball sampling for this research was justified by the small pool of potential participants and the difficulty in recruiting sufficient numbers to reach theoretical saturation. However, this sampling method is not randomised and prone to selection bias [43, 44]. Additionally, because this research explored ATMC students’ experiences in their professional careers, the research only interviewed participants who had passed both the access course and their professional degree, which would be likely to result in a selection bias towards having favourable views of the ATMC.

It is important to note that this study was exploratory in nature and is focused on the experiences of fourteen qualified doctors and one pharmacist who were former students at an ATMC and used this qualification to gain entry to their professional degree. The research is not fully generalisable to all ATMC students, but it does offer insight into ATMC students’ experiences of their course, professional degree and subsequent career and their reflections on these areas.

## Conclusion

Based on the findings of this research, ATMCs are a valuable resource in diversifying the medical profession, which can result in social mobility and can improve patient care by providing a group of doctors who better understand the diverse group of patients that they serve. Further research into ATMCs, alongside adequate funding for those attending them (and for the subsequent professional degrees), coupled with greater publicity and greater geographical availability of providers, are key areas that would improve ATMC participation. This, in turn has the potential to play a key role in efforts to widen participation and improve the quality of care being delivered.

## Electronic supplementary material

Below is the link to the electronic supplementary material.

Supplementary Material 1

## Data Availability

Anonymised interview transcripts are available from SM on reasonable request.

## Abbreviations

ATMCs: Access to Medicine Course/s
WP: Widening participation
GMC: General Medical Council
HE: Higher Education
MSC: Medical Schools Council
QAA: Quality Assurance Agency for Higher Education
CUREC: Central University Research Ethics Committee
BTEC: Business and Technology Education Council
NHS: National Health Service
UCAS: Universities and Colleges Admissions Service
OSCE: Objective Structured Clinical Examination
