# Supplementary material for: Former access to medicine higher education diploma students’ experiences of their diploma course and subsequent professional degree: a qualitative study
Source: BMC Med Educ. 2025 Jul 1;25:902. doi: 10.1186/s12909-025-07399-x (PMC12211710; doi:10.1186/s12909-025-07399-x)
Supplement: Supplementary file 1 — Supplementary Material 1 [file 12909_2025_7399_MOESM1_ESM.docx]

**Supplement 1. Interview questions**

1. Tell me about your qualifications and work background prior to applying to the access to medicine course.
2. Can you describe the events that led to you applying to the access course? How did these influence your decision?
3. How did you first hear about the access course?
4. Tell me about your application to the access course.
5. Tell me about your experience of studying on the access course.
6. Could you describe any positive experiences from, or advantages of, the access course?
7. Could you describe any negative experiences from, or disadvantages of, the access course?
8. How well did the access course prepare you for the process of applying to study medicine at university?
9. When you were at medical school did you feel that you were at an advantage or disadvantage having gained entry via an access course when compared with your peers who were mostly post A levels? Why do you think you felt like that?
10. How did your peers at medical school view your access course?
11. Did you encounter any problems as a consequence of entry into medicine via this route? Can you give me some examples?
12. What advice would you give to someone considering an access course or someone who had completed an access course and had a university place to study medicine?
13. What are your thoughts on widening participation in medicine as a whole?
14. Did the medical school you attended have a foundation year or any other widening participation scheme? If so how do you feel this compared to your access course?
15. Do you think that access to medicine courses offer value for money? What do you think is the evidence for this?
16. Has the route you took into medicine had any positive or negative consequences post qualification? Can you give me examples why you think so?
17. Is there anything else you think I should know or that you would like me to understand better?
